# Forecasting Epidemiological Consequences of Maternal Immunization

**DOI:** 10.1093/cid/ciw557

**Published:** 2016-11-02

**Authors:** Ana I. Bento, Pejman Rohani

**Affiliations:** 1 Odum School of Ecology; 2 Center for the Ecology of Infectious Diseases; 3 Department of Infectious Diseases, University of Georgia, Athens

**Keywords:** pertussis, maternal immunization, routine vaccination, interference, blunting

## Abstract

***Background.*** The increase in the incidence of whooping cough (pertussis) in many countries with high vaccination coverage is alarming. Maternal pertussis immunization has been proposed as an effective means of protecting newborns during the interval between birth and the first routine dose. However, there are concerns regarding potential interference between maternal antibodies and the immune response elicited by the routine schedule, with possible long-term population-level effects.

***Methods.*** We formulated a transmission model comprising both primary routine and maternal immunization. This model was examined to evaluate the long-term epidemiological effects of routine and maternal immunization, together with consequences of potential immune interference scenarios.

***Results.*** Overall, our model demonstrates that maternal immunization is an effective strategy in reducing the incidence of pertussis in neonates prior to the onset of the primary schedule. However, if maternal antibodies lead to blunting, incidence increases among older age groups. For instance, our model predicts that with 60% routine and maternal immunization coverage and 30% blunting, the incidence among neonates (0–2 months) is reduced by 43%. Under the same scenario, we observe a 20% increase in incidence among children aged 5–10 years. However, the downstream increase in the older age groups occurs with a delay of approximately a decade or more.

***Conclusions.*** Maternal immunization has clear positive effects on infant burden of disease, lowering mean infant incidence. However, if maternally derived antibodies adversely affect the immunogenicity of the routine schedule, we predict eventual population-level repercussions that may lead to an overall increase in incidence in older age groups.

Pertussis is an extremely contagious respiratory disease caused by the *Bordetella pertussis* bacterium. Historically, pertussis was considered one of the great microparasitic diseases of childhood, accounting for >4000 annual deaths in the United Stated during the prevaccination era [1]. The introduction of infant vaccination in the 1940s–1950s in industrialized nations was extremely effective in reducing severe disease and mortality [2, 3]. However, the early successes of routine vaccination have given way to resurgence in a number of countries boasting high coverage [2]. The continued circulation of pertussis, both in developing and developed countries, as reported by the World Health Organization [4], has raised serious concerns regarding the effectiveness of current vaccination strategies [3, 5, 6]. Despite the reported high levels of vaccine coverage, there is still substantial pertussis-related morbidity and mortality, especially in infants who have not yet started or completed the primary vaccination schedule [7, 8]. During this window of vulnerability, newborns are susceptible to pertussis infection as a result of contact with infected siblings [9] or parents and other caregivers [2]. In high-vaccine-coverage settings experiencing increasing pertussis incidence, a shift has been observed in the demography of cases, with increasing frequency of cases in teens and young adults [10, 11]. This development has led to the introduction of a booster for adolescents in the United States, Australia, and France, among other countries [12].

The contemporary epidemiology of pertussis is subject to much debate, with a number of candidate explanations put forward for the observed increase in some countries. These range from a true increase to explanations focusing on improved diagnostics and surveillance [3, 4]. Proposed non–mutually exclusive explanations for a true resurgence include the evolution of the bacterium [13, 14], vaccine efficacy [12, 15], waning immunity [16], a drop in coverage due to exemptions [17], and the end of the honeymoon effect [18]. Given the uncertainty in the underlying causes of the resurgence, identifying effective programs for reducing the pertussis burden has proved elusive [6]. The picture is made more blurred in part by the heterogeneity in vaccine use. Many countries, particularly low- and middle-income nations, use whole-cell vaccines, as they are inexpensive and easy to manufacture, while most developed countries have switched to acellular vaccines [4].

A priority for any vaccination policy is the protection of neonates, who are at highest risk of severe morbidity, hospitalization, and death. In principle, if the circulation of a pathogen is successfully reduced through sufficiently high levels of routine vaccination, then even those unvaccinated are afforded protection—a concept known as “herd immunity” [19]. Recent experience, especially in some countries using the acellular pertussis vaccines, has raised doubts regarding the potential success of routine immunization strategies alone. Hence, the attention has shifted toward vaccination programs aimed squarely at protecting infants, including vaccination of likely contacts (“cocooning”) [20] and the immunization of pregnant women, with the 2-fold aim of reducing disease incidence in mothers and the placental transfer of protective antibodies to newborns [4, 21]. Because newborns have an immature immune system [22–29], maternal antibodies (MatAbs) play an important role in their first months of life [30]. Thus, maternal immunization (pregnancy booster) aims to boost specific antibodies in pregnant women and provide direct antenatal protection [9, 12]. Specifically, during the antenatal period, maternal immunization enhances transplacental short-term active transfer of vaccine-induced immunoglobulin G (IgG) [31], with the intention of providing time-limited protection from vaccine-preventable infectious diseases in infants <3 months of age. These high levels of IgG may therefore narrow the vulnerability time window prior to infant routine vaccination [32].

Currently, only 2 vaccines are specifically recommended during pregnancy: influenza and DTaP (diphtheria, tetanus, and acellular pertussis) [33]. Tetanus vaccination of pregnant women has been used for years with great success, significantly reducing neonatal deaths [34]. Unfortunately, this is not always the case; MatAbs may interfere with adaptive immune responses, depending on the ratio of MatAbs and routine vaccination antigen levels (a phenomenon known as epitope masking) [35]. This phenomenon has been documented for some live vaccines (eg, measles), where MatAbs, even in minute quantities, significantly lessen vaccine response [36].

In the first half of the 20th century, studies were conducted on placental transmission of pertussis antibodies as well as on naturally occurring antibodies in young infants to investigate the similarity in characteristics compared to antibodies produced by vaccination [37–39]. Studies using different formulations of the whole-cell vaccine, by Kendrick and colleagues [40], Bradford and Slavin [41], Cohen and Scadron [37], and later by Cashman [39], all showed that the presence of pertussis maternal antibodies in neonates did not appear to have a significant blunting effect (attenuation of pertussis antibody responses) on the subsequent whole-cell immunization routine schedules. These studies were instrumental in establishing the role of natural and vaccine-induced maternal immunity in newborns. However, recent studies on pertussis maternal immunization using acellular vaccines have reported interference effects on infant immune responses to vaccination [42–44]. For instance, Ladhani and colleagues report a 33%–50% reduction in pertussis toxin (PT), filamentous hemagglutinin (FHA), and fimbriae (FIM) titers in infants whose pregnant mothers were immunized [42]. An important epidemiological step remains to establish the population-level and long-term implications of these results, a challenging task given the absence of a serological correlate of protection.

With this in mind, we developed an age-structured transmission model and designed a study to explore the epidemiological outcomes of maternal immunization in various scenarios of routine coverage. In particular, we examined the long-term consequences of potential immunological interference resulting from maternal immunization. Specifically, we assumed blunting resulting in primary vaccine failure (the failure to mount a protective immune response after receiving a dose). We were interested in titrating the implications of maternal immunization and potential blunting on infant incidence (0–2 months of age), the overall mean age of infection across the population.

## METHODS

### Model Formulation

**Figure 1.**
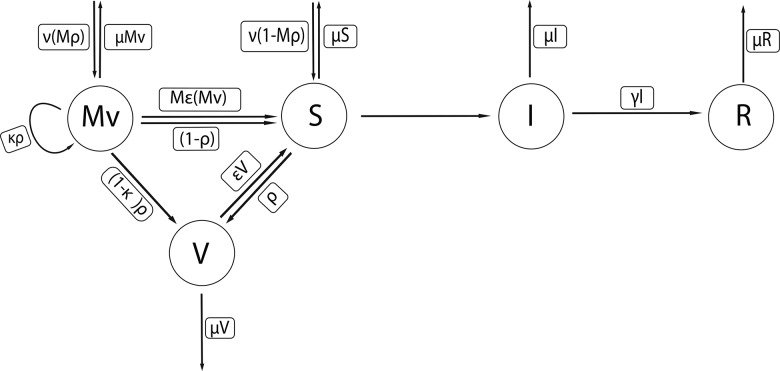
Compartmental transmission model for pertussis with maternal and routine vaccination. Here ν is the per capita birth rate, and μ is the per capita death rate; both were fixed at ν = μ= 1/75 per year. The 5 epidemiological compartments are maternal immunized (Mv), routine vaccinated (V), susceptible (S), infected (I), and recovered (R). Newborns are assumed to be susceptible (S) if their mothers were not vaccinated during pregnancy; otherwise they enter a protected class (Mv). Upon infection, individuals progress to the infectious (I) and ultimately the recovered class (R). Our model is comprised of 18 age classes. Routine infant vaccination occurs at 2, 4, and 6 months of age. It is implemented by moving, with probability ρ (corresponding to the vaccine coverage), those susceptible or maternally vaccinated individuals who age out of their 2-, 4-, or 6-month age categories into the vaccinated (V) class of age 3, 5, or 7 months, respectively. *Mρ* is the maternal immunization coverage, and κ is the interference of maternal-induced antibodies on the routine vaccination (blunting). Individuals in the routine vaccinated class (V) may lose immunity at rate ε, affording protection lasting, on average, 75 years. Similarly; we assume maternally derived immunity wanes at a fixed rate *M*ε. The average infectious period (1/γ) was fixed at 15 days. The model equations and associated parameter values are presented in detail in the Supplementary Materials, Section S1.3 and in Table 1.

We implemented a standard compartmental transmission model [6, 45] for pertussis. The model structure is depicted in Figure 1. Our model consists of 5 epidemiological compartments: maternal immunized (Mv), routine vaccinated (V), susceptible (S), infected (I), and recovered (R). Newborns are assumed susceptible (S) if their mothers were not vaccinated during pregnancy; otherwise they enter a protected class (Mv). Upon infection, individuals progress to the infectious (I) and ultimately the recovered (R) class. The model structure is motivated by previous research that has carried out statistical fitting of transmission models to pertussis incidence reports [45–47]. Our model is comprised of 18 age classes: twelve 1-month infant age classes and 1–4 years, 5–9 years, 10–14 years, 15–19 years, 20–44 years, and ≥45 years. Routine infant vaccination occurs at 2, 4, and 6 months of age to mimic the protective effects afforded following the receipt of 3 doses of pertussis vaccine. It is implemented by moving, with probability ρ (corresponding to the vaccine coverage), those susceptible or maternally vaccinated individuals who age out of their 2-, 4-, or 6-month age categories into the vaccinated (V) class of age 3, 5, or 7 months, respectively. Individuals in the routine vaccinated class may lose immunity at rate ε. Similarly; we assume maternally derived immunity wanes at a fixed rate *M*ε. We emphasize that rates of waning are exponentially distributed, meaning that many will lose immunity faster than the population average.

The average infectious period (1/γ) was fixed at 15 days [3, 18]. The per capita birth and death rates were fixed at ν = μ = 1/75 per year. Finally, we assume that routine vaccination affords protection lasting, on average, 75 years. While there is considerable discussion regarding the nature and duration of pertussis vaccine protection [48–50], we have chosen this particular value to pinpoint the epidemiological impacts of maternal immunization and, in particular, immunological interference. We assumed a basic reproduction number, *R_0_* of 10 [49]. Note that we can quantify an individual-level “vaccine impact” measure, *ϕ*, as defined by Magpantay et al [51], such that eradication requires vaccine uptake to exceed the threshold *ρ*_c_ = (1-1/*R_0_)*/*ϕ*. Thus, under our parameterization, *ρ*_c_ exceeds 1, indicating that eradication via routine immunization is not possible. The model equations and associated parameter values are presented in detail in the Supplementary Materials, Section 1.2 and in Table 1.

**Table 1.** Parameters Used in the Age-Structured Model

Symbol	Parameter	Value
γ	Recovery rate	1/15 days
*C*_ij_	Age-specific contact rate	Supplementary Figure 1
*R*_0_	Reproductive number	10
*ρ*	Routine vaccination	Level (0, 30%, 60%, 98%)
*M*ρ	Maternal immunization	Level (30%, 60%, 90%)
*ϵ*	Waning rate of primary vaccine-derived immunity	75 y^−1^
*M*ε	Waning rate of maternal vaccination immunity	6 mo^−1^
*κ*	Interference of maternal immunity on routine vaccination	Level (10%, 30%, 50%)
*α*, *i* = 1, … 17	Aging rates (y^−1^)	12/1, … 1/4, 1/5, … , 1/20, 1/30
ν	Birth rate	75 y^−1^
μ	Death rate	75 y^−1^

The model consists of 5 compartments: maternal immunized (Mv), susceptible (S), infected (I), recovered (R), and 18 age classes. Here ν is the per capita birth rate (constant), μ is the per capita death rate, α is the rate of aging, ε is the waning rate from routine vaccination, *Mϵ* is the waning rate from maternal immunization, γ is the recovery rate, ρ is the routine vaccination coverage, *Mρ* is the maternal immunization coverage, and κ is the interference of maternal-induced antibodies on the routine vaccination (blunting).

### Contact Network Data and *R_0_* Estimation

Our model incorporated empirical age-specific contact rates from the POLYMOD study in Great Britain [52], corrected for reciprocity as detailed by Riolo and Rohani [6] (Supplementary Figure 1).

We constructed a WAIFW (Who Acquires Infection From Whom) matrix to describe the transmission rate between different age groups (Supplementary Figure 1.1). The basic reproduction number, *R*_0_, for our model was calculated using the next-generation method [53].

### Scenario Analysis

We examined 2 scenarios for the routine vaccination coverage *ρ* = 60% and *ρ* = 98%. Maternal vaccination coverage levels considered were *M*ρ = 30%, 60%, and 90% (Table 1). We incorporated maternal vaccination assuming blunting effects ranging from none, to low (corresponding to 10% primary vaccine failure), to high (equivalent to 50% primary vaccine failure). These blunting effects assume interference manifests as primary vaccine failure, based on serological [42], not clinical data. For every combination of parameters explored, we simulated the model with routine immunization for 200 years before introducing maternal immunization and simulating for a further 200 years (reaching endemic equilibrium). The presented results on age-specific incidence and the overall age at infection were based on model predictions at year 400. Note that our model predictions have not been downsampled to include the effects of potential underreporting and hence represent the “true” pertussis prevalence in our simulated populations.

## RESULTS

### Routine Vaccination and the Window of Susceptibility

**Figure 2.**
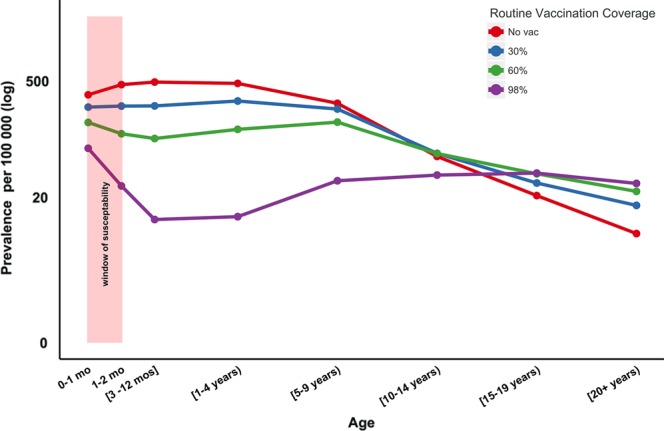
Herd immunity and window of susceptibility. Window of susceptibility is the period between birth and the first dosage of routine vaccination, where infants rely on indirect protection. Levels of routine vaccination coverage (no vaccination [0%], 30%, 60%, 90%, and 98%) and their effects on prevalence and mean age of infection in the different age groups. Mean age of infection is 5.9 years in the absence of vaccination, and 11.6, 19.9, and 38 years for uptake levels of 30%, 60%, and 98%, respectively. Eradication is not attainable, even with our assumption of a vaccine 100% effective, as we also assume it is imperfect in duration, with waning vaccine-derived immunity with a mean duration of protection of 75 years.

In Figure 2, we show that in the absence of routine vaccination, the combined prevalence among infants aged 0–2 months is almost 797 per 100 000, with a peak in the older age groups of 487 per 100 000 among 1- to 5-year-olds. The inclusion of routine immunization at, for example, 60%, reduces infant pertussis prevalence to 277 per 100 000 and not only reduces the peak prevalence in older ages to 161 per 100 000 but also shifts this burden to 5- to 10-year-olds. We emphasize that while a routine coverage level of 98% leads to an order of magnitude reduction in the peak prevalence (40 per 100 000) compared with the vaccine-free scenario, the pertussis burden on the youngest infant groups remains in excess of 106 per 100 000. A useful means of quantifying the impact of vaccination on pertussis circulation is to calculate the mean age at infection [47]. We find this measure to be 5.9 years in the absence of vaccination, and 11.6, 19.9, and 38 years for uptake levels of 30%, 60%, and 98%, respectively.

### Maternal Vaccination Interference Effects on Routine Vaccination (Blunting)

**Figure 3.**
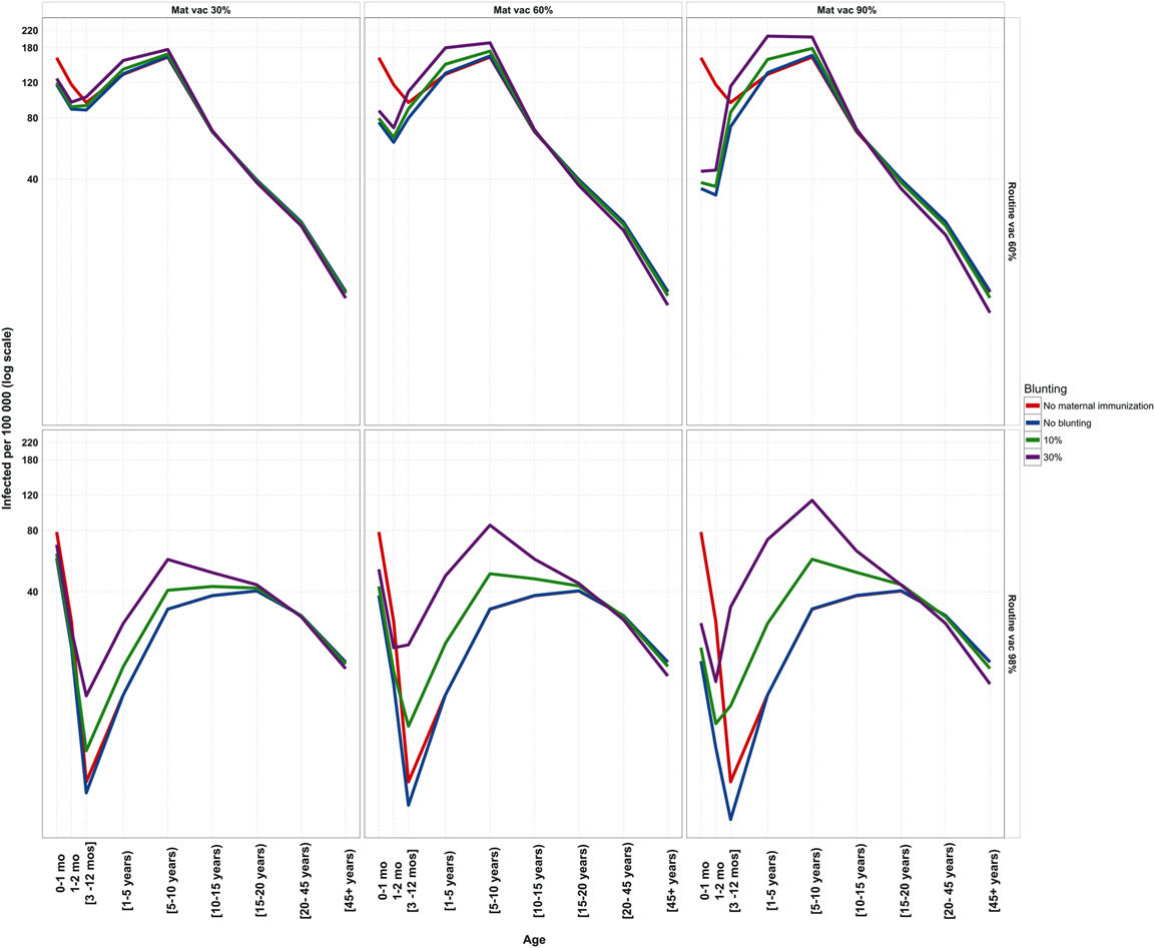
Blunting effects of maternal immunization (Mat vac) on prevalence in both 60% and 98% routine vaccination coverage scenarios. The red line (routine vaccination coverage) is used as a baseline. Blunting (0%, 10%, 30%) here is shown as different levels of primary vaccine failure. Decreases in infant burden (0–3 months) are observed with maternal immunization. Increased blunting effects have consequences in increasing prevalence in the older age classes.

In Figure 3, we illustrate the potential epidemiological consequences of maternal immunization assuming different interference of MatAbs with routine vaccination. The figure demonstrates that maternal immunization successfully reduces the burden of pertussis in the youngest infant age groups. In the absence of blunting, 60% routine and maternal immunization coverage leads to a 50% reduction in prevalence among neonates (0–2 months), compared with the routine vaccination alone. If the presence of MatAbs leads to a 30% risk of vaccine failure, the neonate prevalence is reduced by 43%. However, these gains come at a cost. Under the same scenario, we observe an 18% increase in prevalence among 5- to 10-year olds.

Not surprisingly, when routine coverage reaches 98%, blunting assumes greater importance as the maternal vaccination coverage increases. When maternal immunization coverage is 60%, increasing blunting from 10% to 30% to 50% translates to both increasing prevalence among neonates (57, 71, and 87 per 100 000, respectively) as well as higher transmission to older age groups (5–10 years: 48, 85, and 122 per 100 000, respectively).

Importantly, Figure 3 captures the key tradeoff that may result if MatAbs lead to blunting, or “wasted” routine vaccines (supported by Supplementary Figures 5–8). High maternal immunization coverage (60% or 90%) is shown to be successful in substantially reducing neonatal pertussis. Depending on the strength of blunting, however, this strategy will inevitably also lead to higher prevalence among the older age groups. Assuming 98% routine coverage, 60% prenatal vaccination, and 30% blunting, we observe an almost 4-fold increase in prevalence among 5- to 10-year-olds (122 per 100 000) in comparison with the absence of maternal immunization (32 per 100 000).

Our elasticity analyses are aimed to identify relative marginal gains and losses around specific baseline parameters as maternal immunization and blunting values vary. These analyses show that at 98% routine vaccination coverage with low blunting (around 10%), prevalence is more sensitive to changes in maternal vaccination (rather than changes in blunting levels). In contrast, at high levels of routine and maternal vaccination, incidence is more sensitive to changes in blunting levels (Supplementary Figure 8).

### Mean Age of Infection

**Figure 4.**
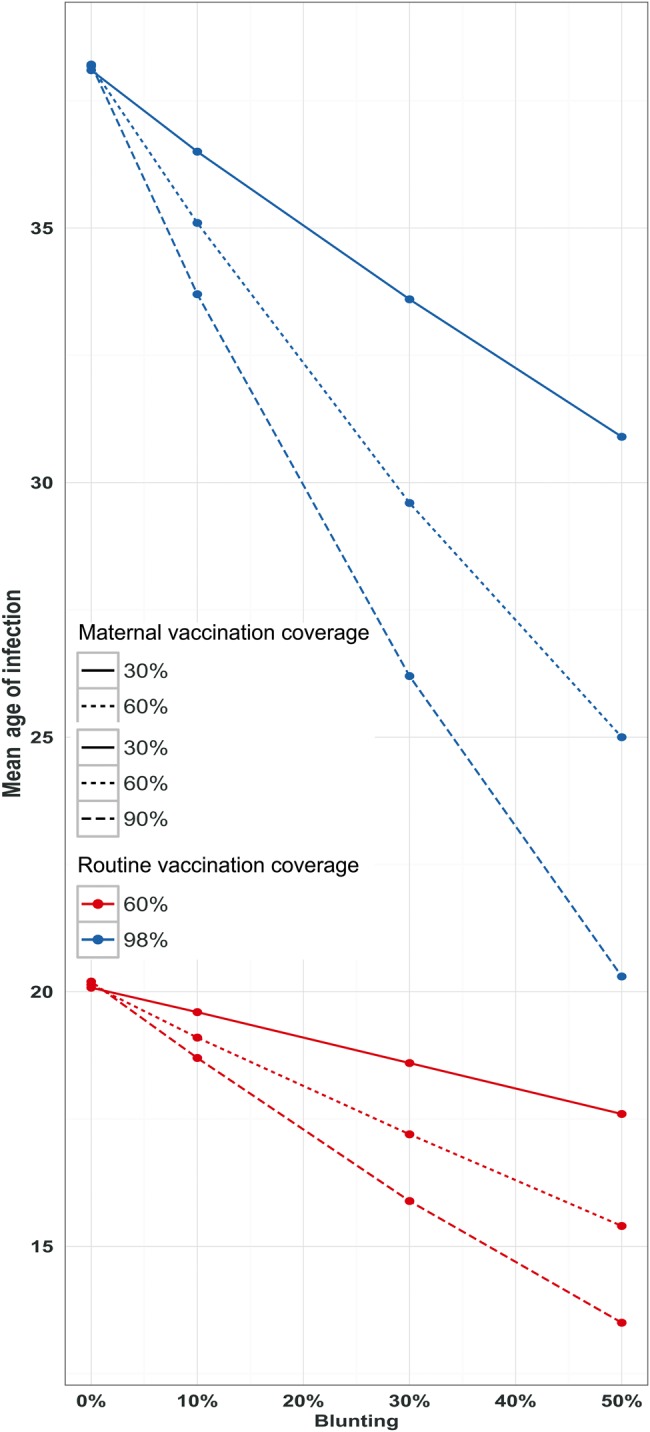
Mean age of infection (years) at different coverage levels of routine vaccination (60% and 98%) and maternal vaccination (30%, 60%, 90%). Blunting (0%, 10%, 30%, 50%) here is shown as different levels of primary vaccine failure. Effects of increasing blunting effects on mean age of infection (years) are quantified.

As with the prevalence, blunting levels increase the overall levels of transmission, naturally leading to a reduction in age at infection. This is illustrated in Figure 4, as well as in Supplementary Figures 5 and 7. In both routine vaccination coverage scenarios, as routine vaccination increases, so does the mean age at infection increases. However, increasing maternal vaccination coverage leads to a reduction in the mean age at infection, with the severity of this effect greatest with very high blunting levels. The effects of blunting are more pronounced with higher maternal vaccination coverage. This is supported by our elasticity analysis (Supplementary Figures 5–8).

## DISCUSSION

Routine infant immunization is intended to simultaneously reduce disease burden and reduce transmission, thereby providing indirect protection to unvaccinated individuals in the population [19]. In the case of pertussis, with ongoing concerns about the protectiveness of contemporary (acellular) vaccines and the general increase in incidence reported in a number of countries, reliance on indirect protection of infants in the first 2 months of life who are too young to be immunized is unwise. For a significant reduction of the infant pertussis burden, attention has turned toward safe immunization strategies targeting early life, in particular through maternal vaccination. Its purpose is to vaccinate pregnant mothers in the third trimester with the aim of directly protecting the mother and, through placental transfer of antibodies, the neonate. In the Netherlands, a cost-effectiveness analysis study by Westra and colleagues [54] showed that maternal immunization was found to be efficient in lowering infants' incidence. Recently, maternal pertussis immunization, under an acellular vaccination regime, has been recommended in the United States and in the United Kingdom (vaccination made available to all women in their 27- to 36-week period of each pregnancy when IgG transplacental transfer is at its highest) [5, 30], as part of a plethora of disease control strategies, not only for the mother and the developing fetus but also to the newborn, to reduce the burden of pertussis at population level [55]. Similar recommendations were adopted to protect newborns in Argentina, Belgium, Israel, New Zealand, Uruguay, Costa Rica, Mexico, Panama, and Brazil [56, 57].

However, one possible issue that has been raised regarding the use of maternal immunization is that antibodies produced as a result of vaccination during pregnancy will attenuate or interfere with the elicitation of an immune response to the primary routine vaccination, an effect often referred to as blunting. Several studies at the end of the 20th and, more recently, in the 21st century have examined antibody responses in individuals who received DTP (whole-cell vaccine) as part of the routine schedule and who had high levels of maternally induced antibodies. These individuals had a reduced PT antibody response, which determines the severity of pertussis in unprotected newborns [57]. In a more recent study, Ladhani and colleagues [42] compared historical cohort data where mothers had not been vaccinated while pregnant with infants whose mothers were vaccinated. They found that infants had high pertussis antibodies concentrations prior to first routine dose (acellular vaccine), with PT significantly increasing postimmunization. However attenuation was noted where FHA levels were significantly lower postvaccination (see also [43, 44]). The mechanisms of protection need to be better understood; protection against disease might be stronger and longer than predicted by antibody titers alone [58].

Currently, because there are no known serologic correlates of protection to pertussis [59], there is uncertainty regarding the nature, degree, and duration of vaccine immunity, and by implication the clinical consequences of these serological observations. Our study quantifies the potential effects of interference by maternal immunization assuming the pattern of antibody responses observed by Ladhani et al [42] results in primary routine vaccination failure (see also [43, 44]). If changes in antibody responses do not adversely affect protection afforded by routine vaccination, the epidemiological benefits of maternal immunization are clear (Figure 3; Supplementary Figures 2 and 3). As we show in Figures 3 and 4, neonates benefit from maternally induced immunity, leading to a reduction in prevalence. Our results complement the conclusions drawn by Terranella et al [60], who demonstrated that Tdap maternal vaccination would lead to fewer cases, hospitalizations, and deaths compared with other control strategies such as other cocooning strategies (eg, postpartum vaccination). We anticipate that similar findings would result if blunting effects are transient.

However, our modeling results anticipate a potential downstream risk associated with MatAbs interference—namely, an eventual increase in prevalence among older age groups. Simulations indicate these effects may take a decade or more to be made manifest. The magnitude of these repercussions at the population level is dependent on the severity of MatAbs interference (Figure 4).

Ultimately, the robustness of our modeling conclusions will be determined by additional empirical information. We hope additional light will be shed on this issue from the ongoing randomized, double-blind study of maternal pertussis vaccines in Canada and a recently completed US trial. These studies will help clarify the possible blunting effects of maternal antibodies on infant immune response to routine vaccination scheduling and, crucially, whether these effects may simply correspond to an effective reduction in vaccine “take” (as we have assumed here), or perhaps a failure in vaccine “degree” or the duration of protection [61, 62]. Ultimately, long-term clinical data from settings such as the United Kingdom, where maternal immunization has been practiced since 2012, will be necessary to test the veracity of the epidemiological predictions of our model, specifically the potential increases in incidence among older age groups.

We submit that empirically validated transmission models may play a role in answering a number of remaining policy questions—for example, whether maternal immunization is most effective as standard policy or more suitable as a measure during an outbreak. Similarly, it remains to be seen whether MatAbs interference is observed when the routine schedule includes at least 1 dose of the whole-cell vaccine, as pertinent to low- and middle-income countries. Finally, our results are predicated on the empirically demonstrated assumption that maternally derived antibodies persist in infants for an average of 6 months [42, 63]. A potentially fruitful avenue for further research is optimizing the routine schedule, such that the first dose is administered perhaps at a later age, depending on the blunting effects of lingering maternal antibodies.

## Supplementary Data

Supplementary materials are available at http://cid.oxfordjournals.org. Consisting of data provided by the author to benefit the reader, the posted materials are not copyedited and are the sole responsibility of the author, so questions or comments should be addressed to the author.

Supplementary Data
