# Erratum

**DOI:** 10.1093/cid/ciw853

**Published:** 2017-03-04

Four errors appeared in an article published with the 1 December 2016 supplement (Bento AI, Rohani P. Forecasting Epidemiological Consequences of Maternal Immunization. Clin Infect Dis 2016; 63[suppl 4]:S205–12). First, the first sentence of the fourth paragraph should refer to the vaccine “Tdap” (not DTaP). Second, the Supplementary Data file has been updated to reflect the correct author listing. Third, in the legend of Figure 2, the level of vaccination coverage does not include 90%. Last, in the legend for Figure 4, there should be no 50% blunting.

The authors regret these errors.

## Supplementary Material

ciw557_Erratum_SuppClinInfDis

