# Supplementary material for: Forecasting Epidemiological Consequences of Maternal Immunization
Source: Clin Infect Dis. 2016 Nov 2;63(Suppl 4):S205–12. doi: 10.1093/cid/ciw557 (PMC5106624; doi:10.1093/cid/ciw557)
Supplement: Supplementary Data [file supp_63_suppl-4_S205__index.html]

Supplementary Data 

# Forecasting Epidemiological Consequences of Maternal Immunization

## Supplementary Data

Supplementary Data

- Supplementary Data - Pdf file
