# Supplementary material for: Erratum
Source: Clin Infect Dis. 2017 Mar 4;64(9):1298. doi: 10.1093/cid/ciw853 (PMC5399933; doi:10.1093/cid/ciw853)
Supplement: ciw557_Erratum_SuppClinInfDis [file ciw853_suppl_ciw557_Erratum_SuppClinInfDis.pdf]

Forecasting epidemiological consequences of maternal immunization: Supplementary File

1.Odum School of Ecology, University of Georgia, Athens, GA, USA

2.Center for the Ecology of Infectious Diseases, University of Georgia, Athens, GA, USA

3.Department of Infectious Diseases, University of Georgia, Athens, GA, USA

Ana I. Bento[1,2]; Pejman Rohani[1,2,3]†*

S1 Supplementary methods

S1.1 Model formulation and parameter values

We implemented an age-structured, compartmental model of pertussis transmission, using previous described models as reference [1-3]. The model is an extension of the classic SIR model, it incorporates both maternal and routine vaccination. Susceptibles individuals may become infected on contact with infected individuals. Infected individuals upon recovery move to the recovered class R. To account for possible differences between infection and vaccine-derived immunity, vaccinated individuals are explicitly modelled (V). Indviduals born from a mother who was vaccinated while pregnant are explicitly modelled as Mv. Maternal vaccination is modelled with a waning rate $M\epsilon(Mv)$. (see table S1 for parameter model parameter values)

When modelling routine vaccination in the absence of vaccine induced maternal antibodies, for simplicity, we only consider one type of routine vaccination failure- Waning - where immunized individuals lose their immunity and become susceptible at a rate ϵV for vaccine-derived immunity.

When modelling interference effects of maternal vaccination on routine vaccination, we consider it to be primary vaccine failure of routine vaccination due to interference of vaccine induced maternal antibodies. Where individuals fail to mount an immune response remaining in the Mv category for the duration of afforded protection until they move to the S class. (see table S1 for parameter model parameter values). This is modelled a fraction of the Mv not being moved to V.

*Corresponding author: anabento@uga.edu

†Alternative corresponding author: rohani@uga.edu

Overall, the model consists of 18 age groups, labelled i = 1, . . . , 18. The age groups are as follows, 12 1-month infant age classes and the following age categories for the older age groups (1-4), (5-19), (10-14), (15-19), (20-44), 45+. We include the newborn classes to account for the maternal and routine immunization effects. Routine infant vaccination occurs at 2,4, and 6 mo of age in order to mimic the protective effects afforded following the receipt of three doses of pertussis vaccine. Aging occurs continuously, at rates $\alpha_i = \frac{1}{\Delta a_i}$ yr^{-1}, where Δa_i is the age span in age group i.

To model the effect of the maternal vaccination, a fraction of the newborns (age class 1) start off in the Mv compartment. To model the effect of routine vaccination, a fraction of the individuals either from S or Mv are moved to V on aging from 1 to 2 months of age.

The model is described by the system of differential equations below and was integrated numerically.

S1.2 Contact network and calculating the reproduction number R_0

Our model used Great Britain empirical age-specific contact rates from the POLYMOD study [4], corrected for reciprocity [5]. Where, C_{ij} is the average number of daily contacts in Mossong *et al.* [4]) reported by a "contacter" of age group i with "contactees" of age group j (individuals are classified into 0-1 year and then (1-4), (5-19), (10-14), (15-19), (20-44), 45+ age groups so that $1 \leq (i, j) \leq 18$). N_i represents individuals in age group i in the population. $E_{ij} = N_i C_{ij}$. is the average total number of contacts between age groups i and j. The matrix E = (Eij) was transformed to become symmetric: E $\rightarrow \frac{1}{2}$ (E+E^{T}). Thus, the individual average number of daily contacts between age groups i and j, corrected for reciprocity: $\forall(i, j), Cij = \frac{E_{ij}}{N_i}$. We augmented the age class 0-1 years of age into monthly classes by replicating the (0-1y) 11 times making sure the sum of contacts is the same as before. We assumed that individuals in the 0–1 y age group have identical number of contacts: $\forall(j), augmentedC_{1j} = augmentedC_{2j} = ...augmentedC_{11j} = C_{1j}$. R_0 is the basic reproduction number in the absence of vaccination ($\rho = 0$). Calculations used the age classes defined above (i=1, . . . , 18) and a constant birth rate $\nu = \frac{1}{75}$ yr^{-1}, at disease free equilibrium in the absence of vaccination ($\rho = 0$): $\forall_i$, $S_i = N_i$, $V_i = 0, where R_i = 0$ is the dominant eigenvalue of the next-generation matrix [6, figure S1].

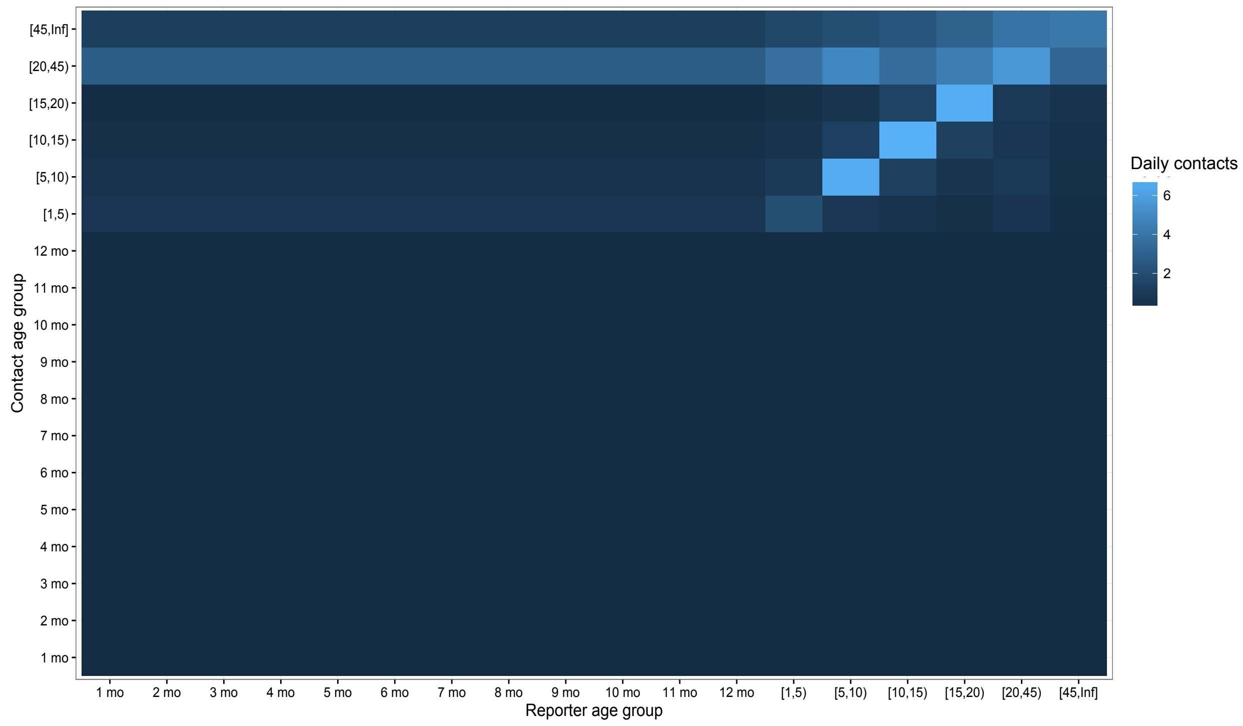

Figure S1. Age-specific contact matrix used in the simulations.

S1.3 Model equations

For newborns up to 1 month old (i=1):

(1)

$$\frac{dS_1}{dt} = \nu(1 - M\rho)N - \lambda_1 S_1 + M\epsilon - \mu S_1 - \alpha_1 S_1$$

(2)

$$\frac{dI_1}{dt} = \lambda_1 S_1 - \mu I_1 - \alpha I_1$$

(3)

$$\frac{dR_1}{dt} = \gamma I_1 - \mu R - \alpha_1 R_1$$

(4)

$$\frac{dMv_1}{dt} = \nu M\rho N - M\epsilon - \mu Mv_1 - \alpha_1 Mv_1$$

For newborns 2 month old (i=2):

(5)
$$\frac{dS_2}{dt} = -\lambda_2 S_2 + M\epsilon Mv_2 - \mu S_2 - \alpha_2 S_2 + \alpha_1 S_1(1-\rho) + \epsilon V_2$$

(6)
$$\frac{dI_2}{dt} = \lambda_2 S_2 - \mu I_2 - \alpha 2 I_2 + \alpha 1 I_1$$

(7)
$$\frac{dR_2}{dt} = \gamma I_2 - \mu R_2 - \alpha_2 R_2 + \alpha 1 R_1$$

(8)
$$\frac{dMv_2}{dt} = -M\epsilon Mv_2 - \mu Mv_2 - \alpha_2 Mv_2 + \alpha_1 Mv_1(1-\rho)\kappa$$

(9)
$$\frac{dV_2}{dt} = -epsilon V_2 - \mu V_2 - \alpha V_2 + \alpha_1 Mv_1 \rho(1-\kappa)$$

For infants age 3 to 6 months old (i = 3, . . . , 6)

(10)
$$\frac{dS_i}{dt} = -\lambda_i S_i - M\epsilon V_i - \mu S_i - \alpha S_i + \alpha S_{i-1} - \epsilon V_i$$

(11)
$$\frac{dI_i}{dt} = \lambda_i S_i - \mu I_i - \alpha i I_i + \alpha i - 1 I_{i-1}$$

(12)
$$\frac{dR_i}{dt} = \gamma I_i - \mu R_i - \alpha R_i - \alpha i R_i + \alpha i - 1 R_{i-1}$$

(13)
$$\frac{dMv_i}{dt} = -M\epsilon Mv_i - \mu Mv_i - \alpha_i Mv_i + \alpha_{i-1} Mv_{i-1}$$

(14)
$$\frac{dMv_i}{dt} = -\epsilon V_i - \mu V_i - \alpha V_i + \alpha_{i-1} Mv_{i-1}$$

For individuals 7 mos to 45+ years (i = 7, . . . , 18)

(15)
$$\frac{dS_i}{dt} = -\lambda_i S_i - M\epsilon V_i - \mu S_i - \alpha S_i + \alpha S_{i-1} - \epsilon V_i$$

(16)
$$\frac{dI_i}{dt} = \lambda_i S_i - \mu I_i - \alpha i I_i + \alpha i - 1 I_{i-1}$$

(17)
$$\frac{dR_i}{dt} = \gamma I_i - \mu R_i - \alpha R_i - \alpha i R_i + \alpha i - 1 R_{i-1}$$

(18)
$$\frac{dMv_i}{dt} = 0$$

(19)
$$\frac{dMv_i}{dt} = -\epsilon V_i - \mu V_i - \alpha V_i + \alpha_{i-1} Mv_{i-1}$$

Here ν is the birth rate (constant), μ is the death rate, α is aging, ϵ is the waning rate from routine vaccination $M\epsilon$ is the waning rate from maternal immunization, γ is the recovery rate, ρ is the vaccination coverage, $M\rho$ is the maternal immunization coverage, and κ is the interference of maternal induced antibodies on the routine vaccination (blunting).

The force of infection in age group i = 1, . . . , 18 is defined as

$$\lambda_i = \sum_j qC_{i,j}\frac{I_j}{N_j}$$

here, q is the probability of infection given exposure in each age group. We fixed the q parameter so R_0 was in agreement with previous pertussis models [13].

S1.4 Calculating Mean age of infection

In the presence of vaccination interventions, mean age of infection (A) is dependent on the effective reproductive number (R_p)[6]. We expect mean age of infection to decrease as R_p increases. In our age structured model, we defined mean age of infection as:

$$A = \sum_i \frac{I}{N} t_{age}$$

where, I is the number of infected at each age class, N is the total number of individuals in the population and t_{age} is the time spent in each age class.

S2 Supplementary results

S2.1 Sensitivity analysis

Maternal immunization effects on infant cases in a scenario where there is no interference with immune response to routine vaccination

In the absence of interference effects (figure S2), unsurprisingly, as we increase maternal immunization coverage, infant burden is alleviated. This is particularly evident in the first months of life when neonates, in a scenario with no maternal immunization rely on herd immunity to indirectly protect them from coming into contact with infected individuals.

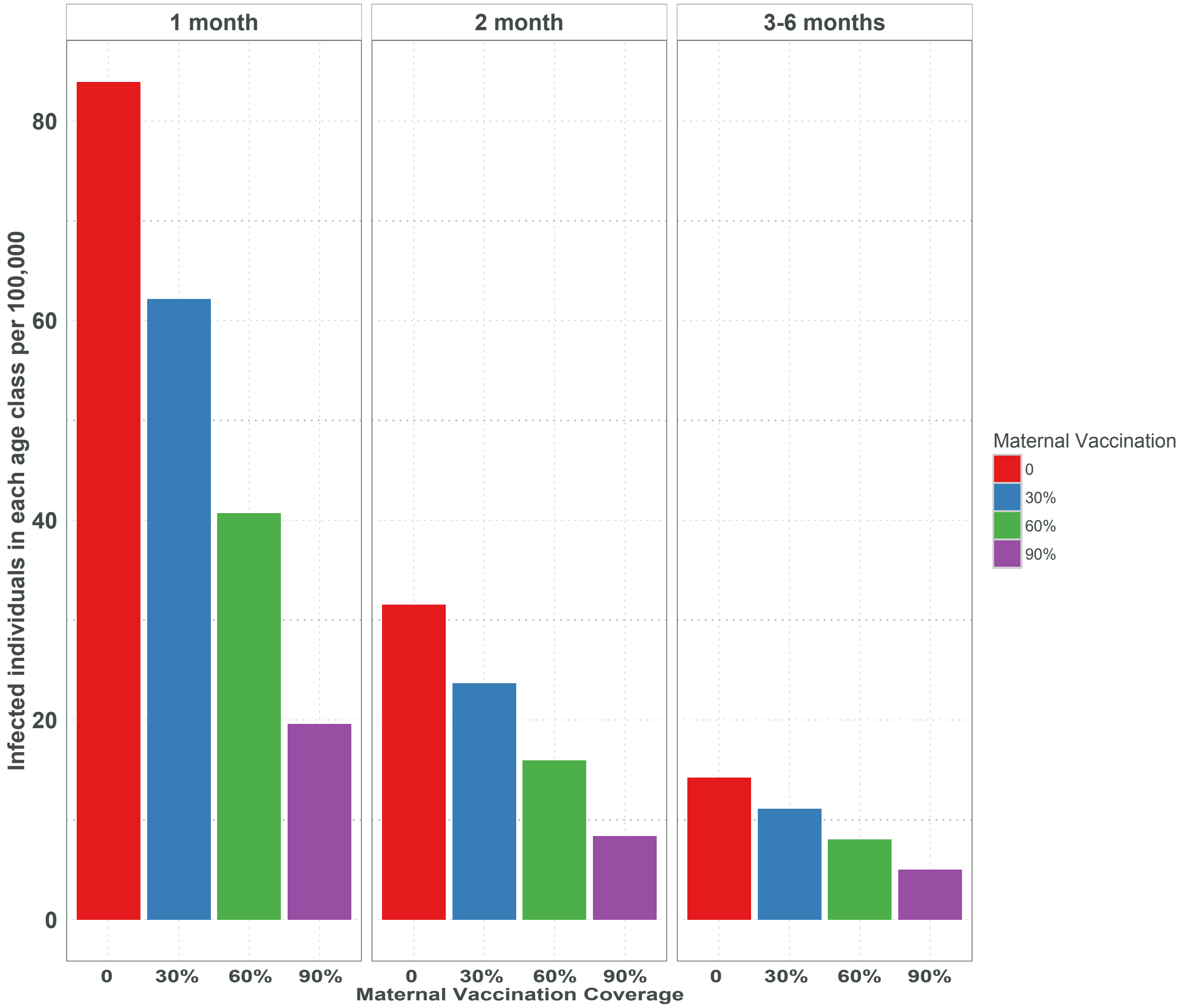

Figure S2. Maternal immunization coverage effects on infant cases (0- 6 months) at a 98% routine

vaccination (where there is waning of vaccine induced immunity), in this scenario there is no blunting.

Interference effects on routine vaccination (blunting) in a scenario with differing levels interference with immune response to routine vaccination

Figure S3 illustrates the potential effects of increasing interference of maternal immunization on routine vaccination. As in figure 3, at 60% routine vaccination, with increasing maternal vaccination coverage and low level of blunting (10% primary vaccine failure) we see a decrease in susceptibles in the younger age classes. We also see an increase in susceptibility in older age classes. As blunting increases, there is an increase in susceptibles, specifically in the 3 months to 5 years age classes. At 98% routine vaccination coverage, as the maternal vaccination coverage increases, the potential effects of blunting become more striking in all age classes, with an increase in susceptibles in all age classes except in the neonates. When comparing between routine vaccination coverage levels, at higher coverage, nor surprisingly, susceptibility decreases, with blunting effects being more pronounced especially in the younger age classes, due to the increased transmission.

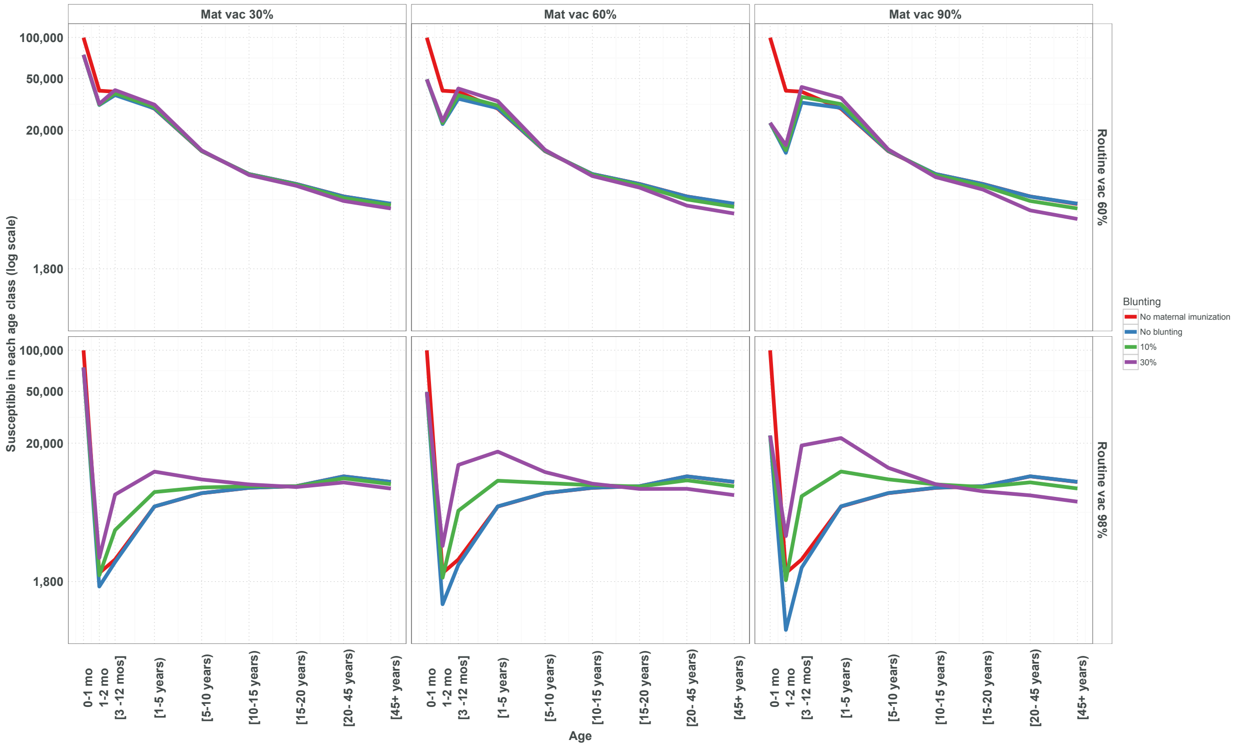

Figure S3.Blunting effects of maternal immunization susceptible individuals at each age group. The red line (routine vaccination coverage) is used as a baseline. Maternal immunization without

blunting effects and with blunting (10, 30%) here is shown as different levels of primary vaccine failure. Blunting with 50% not shown here (shown as part of the elasticity analysis S2.2)

Figure S4 illustrates that our modeling results anticipate a downstream risk associated with MatAbs interference, namely an eventual increase in prevalence among older age groups. Simulations indicate these effects may take a decade or more to be made manifest. We show that the magnitude of these repercussions at the population level is dependent on the severity of MatAbs interference.

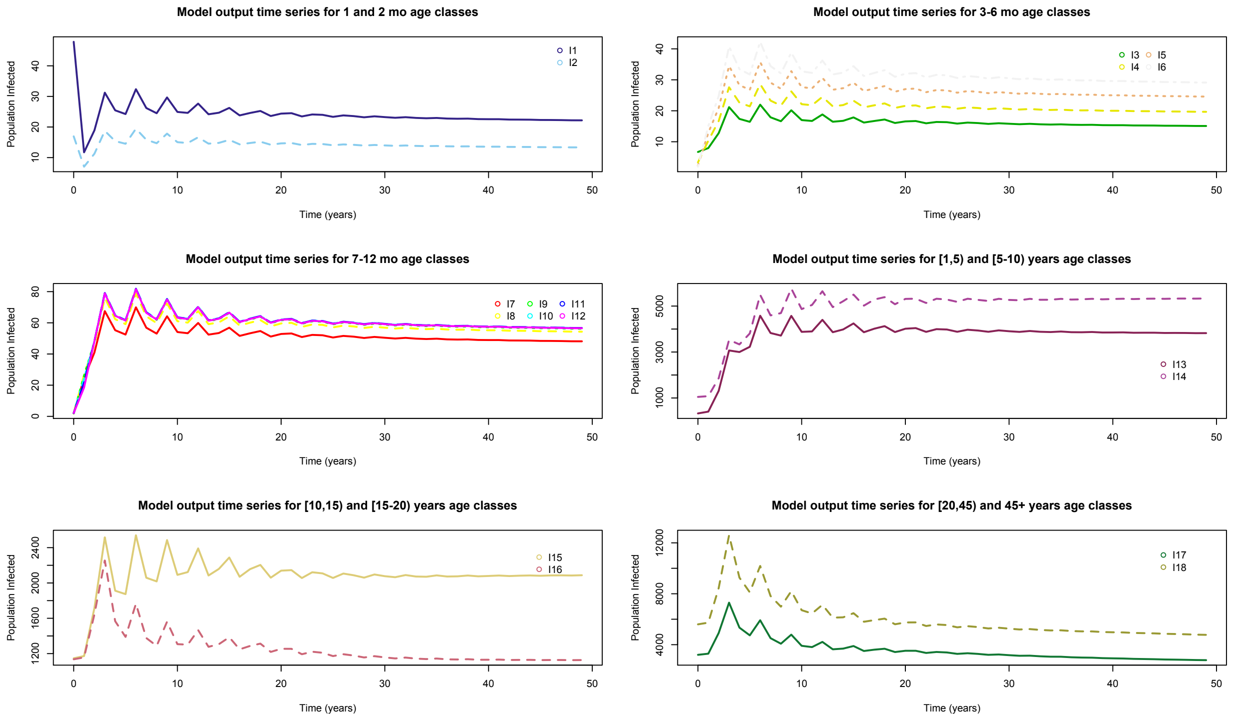

Figure S4. Model output time series (200 years). Downstream risk associated with MatAbs interference in each age category, after implementaion of maternal immunization the model runs for 200 years.

S2.2 Elasticity analysis

By allowing both maternal immunization and blunting (the measure of interference of maternally derived immunity on immune response to routine vaccination) to vary by small amounts, we measured the relative response of those "perturbations", in a deterministic way, on both mean age of infection and mean infant incidence (0-3 mo). By perturbing both interference levels and maternal vaccination coverage we quantified the effects on mean age of infection and mean infant

incidence, we quantified the effects on mean age of infection and mean infant incidence. Here we show the results for a scenario where routine vaccination coverage is 60%. By allowing both maternal immunization coverage and blunting (the measure of interference of maternally derived immunity on immune response to routine vaccination) to vary by relatively small amounts we measured the relative response of those "perturbations" in a deterministic way on both mean age of infection and mean infant incidence (0-3 mo). The results for 98% coverage are in the main text.

Routine Vaccination 60%

1. Mean age of infection

We compare the effects of small changes in blunting and maternal immunization on mean age at infection. At lower maternal vaccination coverage, the effects of increasing blunting are almost negligible. Mean age of infection is more sensitive to change sin blunting at higher levels of maternal immunization coverage.

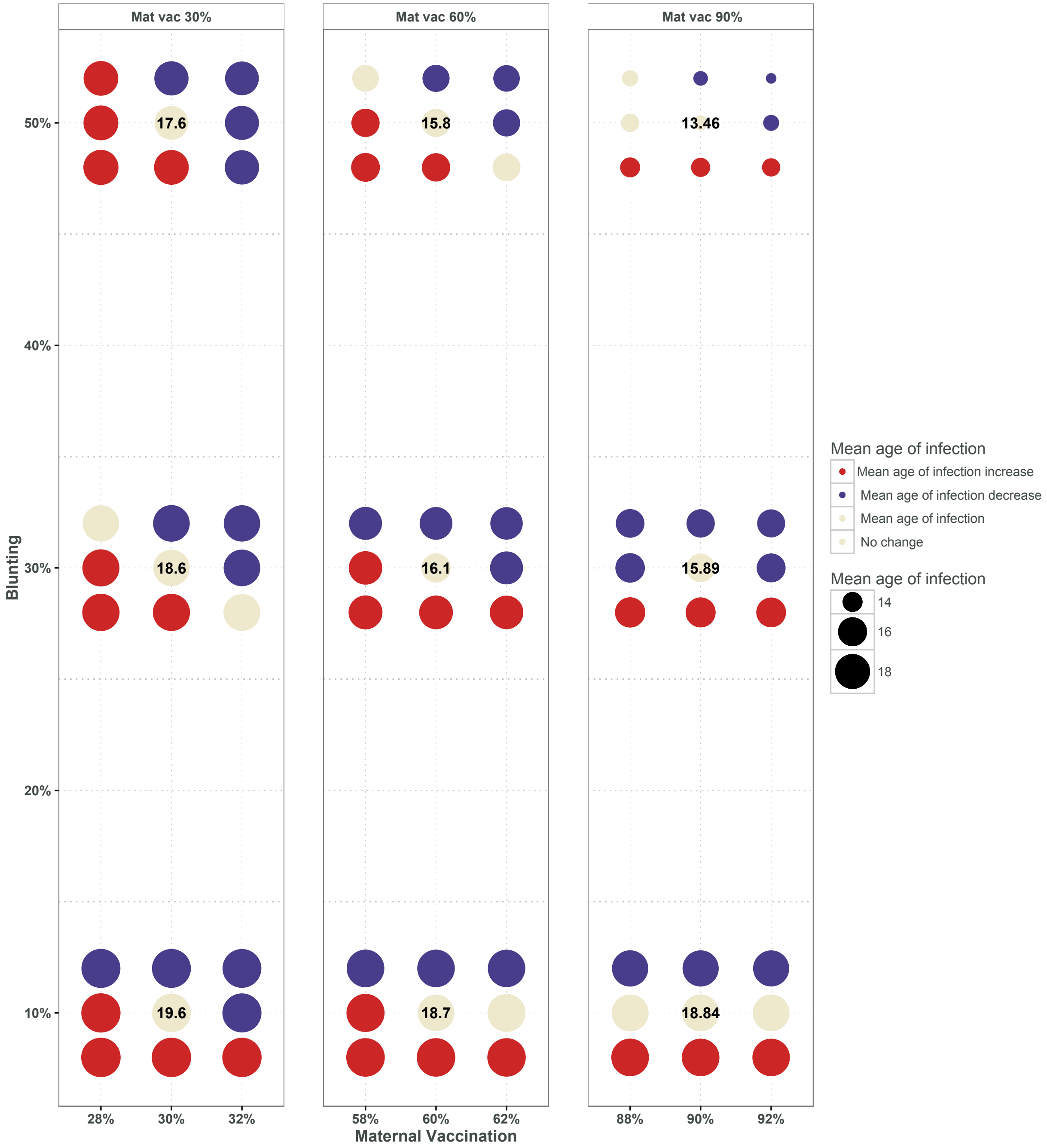

Figure S5. Mean age of infection. Effects of small changes in blunting levels (primary vaccine failure) and maternal immunization, at a 60% routine vaccination coverage. In pale yellow is the baseline level and in red and blue are the effects of those small changes on mean age of infection.

2. Mean infant incidence

The results from our elasticity analyses show that, at 60% routine vaccination coverage, not surprisingly, infant incidence decreases with increase of maternal immunization coverage (Figure S6). We compare the effects of small changes in blunting and maternal immunization on mean age at infection. At lower maternal vaccination coverage, the effects of increasing blunting are almost negligible. Mean infant incidence is more sensitive to changes in blunting at higher levels of maternal immunization coverage. Mean infant incidence decreases as maternal immunization coverage increases.Incidence is mostly affected by maternal immunization coverage (row comparisons). Blunting levels changes have a reduced effect on incidence (column comparison), but those effects are less noticeable at lower levels of maternal immunization coverage.

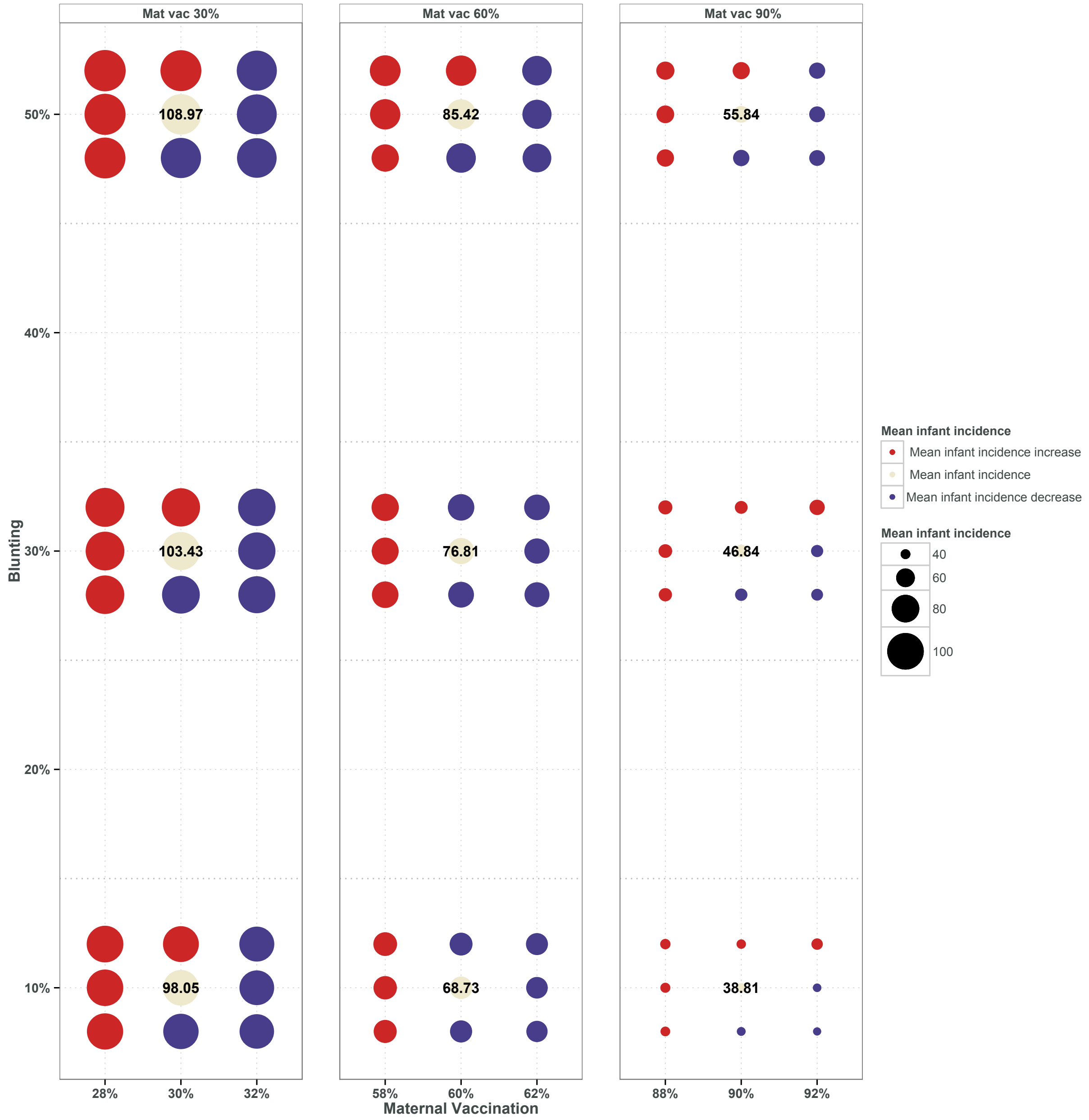

Figure S6. Mean infant incidence (0-3 months) with 60% routine vaccine coverage. Effects of small changes in blunting levels (primary vaccine failure) and maternal immunization, at a 60% routine vaccination coverage. In pale yellow is the baseline level and in red and blue are the effects of those small changes.The size of the bubbles indicate the size of effect and the colour change indicate a decrease (blue) or an increase in mean infant incidence at middle points of combinations of blunting effects and maternal immunization coverage.

Routine Vaccination 98%

1. Mean age of infection

Mean age of infection increases in comparison with the scenario of 98% routine immunization coverage. We now compare the effects of small changes in blunting and maternal immunization on mean age at infection at 98% routine coverage. At lower maternal vaccination coverage, the effects of increasing blunting are almost negligible. Mean age of infection is more sensitive to change sin blunting at higher levels of maternal immunization coverage.

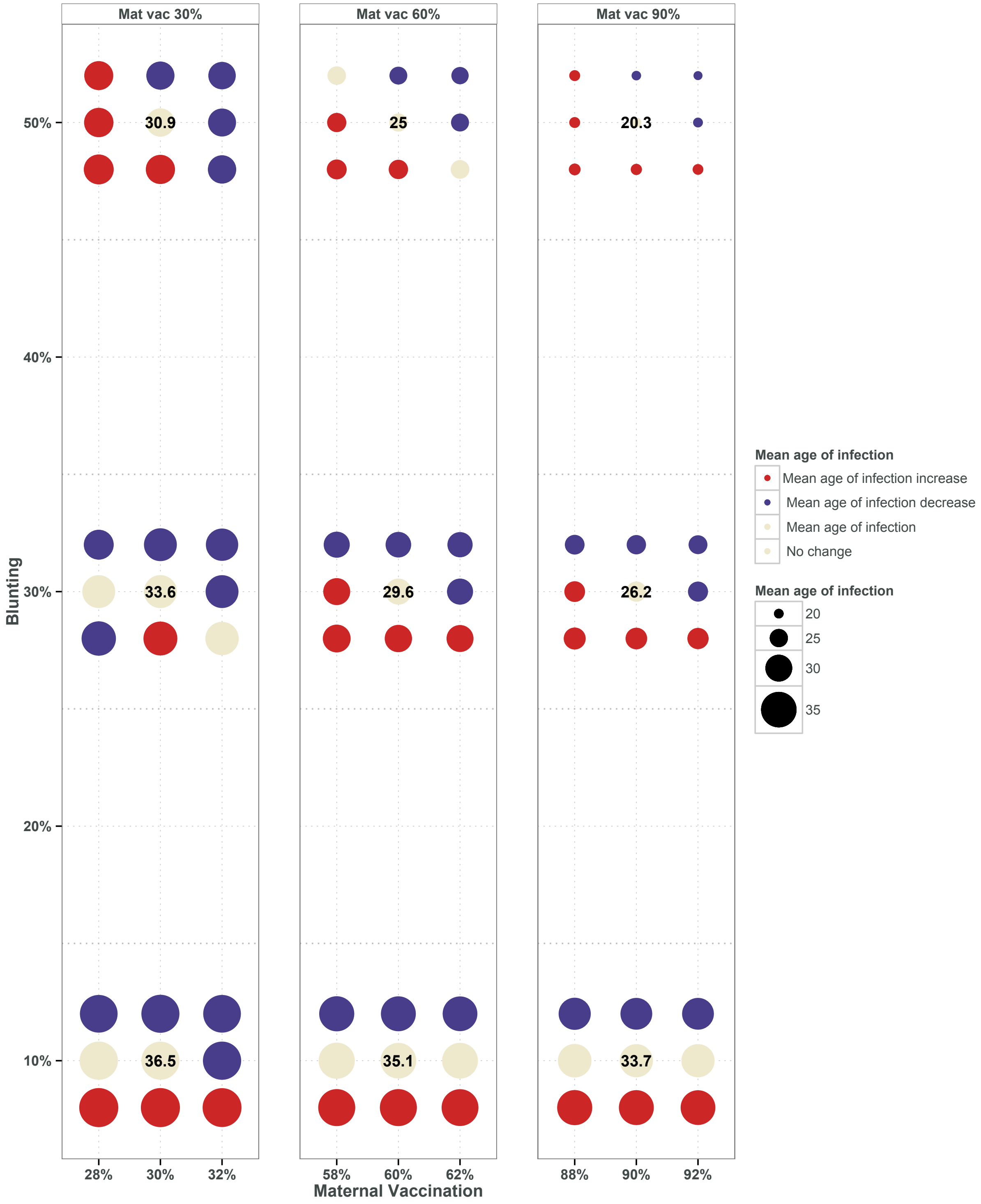

Figure S7. Mean age of infection. Effects of small changes in blunting levels (primary vaccine failure) and maternal immunization, at a 98% routine vaccination coverage. In pale yellow is the baseline level and in red and blue are the effects of those small changes on mean age of infection.The size of the bubbles indicate the size of effect and the colour change indicate a decrease (blue) or an

increase in mean age of infection at middle points of combinations of blunting effects and maternal immunization coverage.The size of the bubbles indicate the size of effect and the colour change indicate a decrease (blue) or an increase in mean age of infection at middle points of combinations of blunting effects and maternal immunization coverage.

2. Mean infant incidence

The results from our elasticity analyses show that, at 98% routine vaccination coverage, not surprisingly, infant incidence decreases with increase of maternal immunization coverage (Figure S8). Mean infant incidence decreases in comparison with the 60% routine vaccination coverage.like with the 60% routine coverage scenario, incidence is mostly affected by maternal immunization coverage (row comparisons). Blunting levels changes have a reduced effect on incidence (column comparison (Figures S6 and S8), but those effects are less noticeable at lower levels of maternal immunization coverage. We compare the effects of small changes in blunting and maternal immunization on mean age at infection. At lower maternal vaccination coverage, the effects of increasing blunting are almost negligible. Mean infant incidence is more sensitive to changes in blunting at higher levels of maternal immunization coverage. Mean infant incidence decreases as maternal immunization coverage increases. When blunting is low (8-12%), a small decrease in maternal vaccination, results in an increase in incidence. At high levels of maternal vaccination, incidence is very sensitive to changes in blunting levels even when not changing maternal immunization coverage (Figure S8).

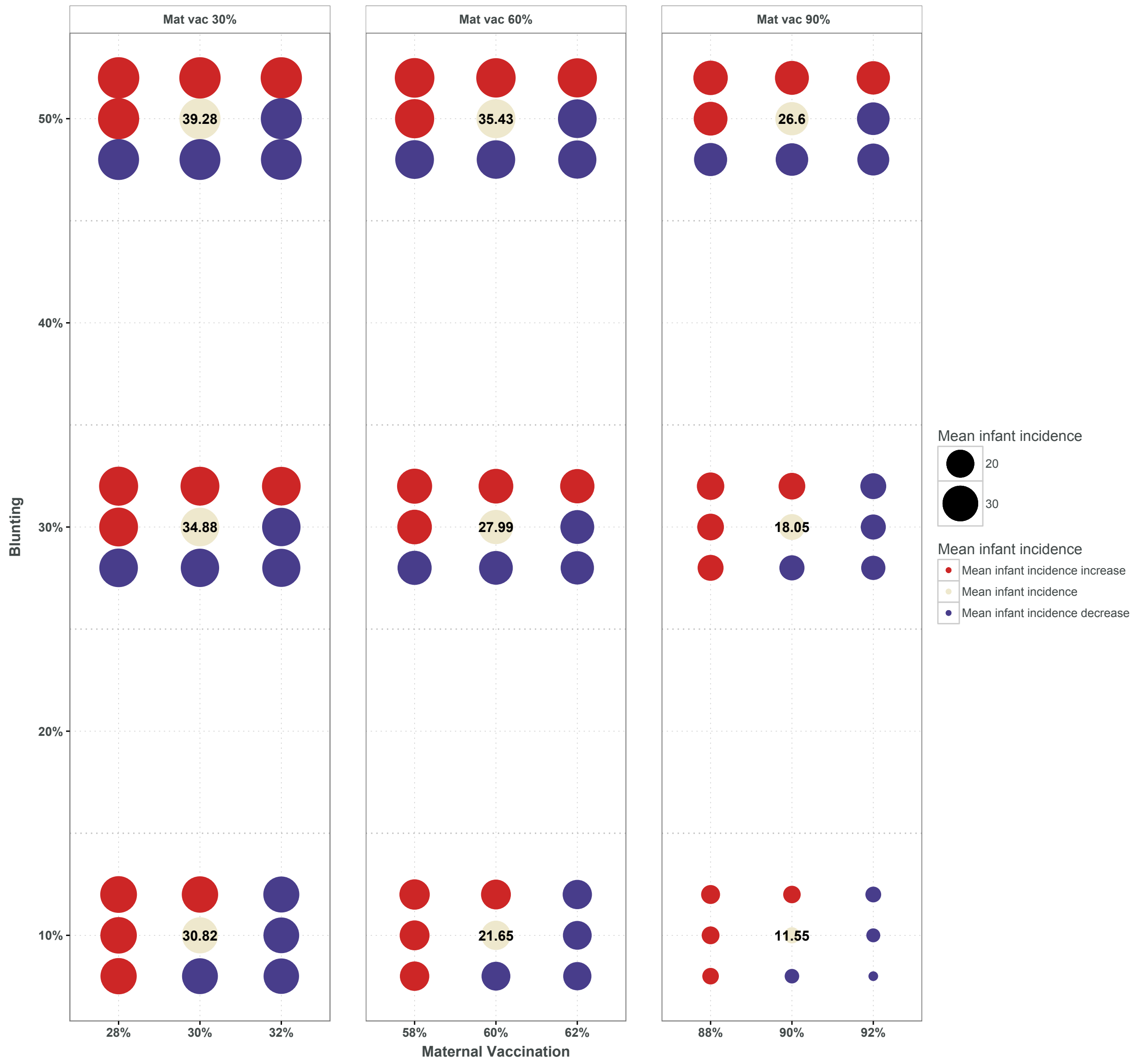

Figure S8. Mean infant incidence with 98% routine vaccine coverage. Effects of small changes in blunting levels (primary vaccine failure) and maternal immunization, at a 98% routine vaccination coverage. In pale yellow is the baseline level and in red and blue are the effects of those small changes.The size of the bubbles indicate the size of effect and the colour change indicate a decrease (blue) or an increase in mean infant incidence at middle points of combinations of blunting effects and maternal immunization coverage.

References

1. Wearing, H. J. & Rohani, P. Estimating the duration of pertussis immunity using epidemiological signatures. PLoS Pathog 5, e1000647 (2009).
2. Rohani, P., Zhong, X. & King, A. A. Contact network structure explains the changing epidemiology of pertussis. Science 330, 982–5 (2010).
3. Blackwood, J. C., Cummings, D. A. T., Broutin, H., Iamsirithaworn, S. & Rohani, P. Deciphering the impacts of vaccination and immunity on pertussis epidemiology in Thailand. Proc Natl Acad Sci, USA 110, 9595–600 (2013).
4. Mossong, J. et al. Social contacts and mixing patterns relevant to the spread of infectious diseases.PLoS Med 5, e74 (2008).
5. Riolo, M. A., King, A. A. & Rohani, P. Can vaccine legacy explain the British pertussis resurgence? Vaccine 31, 5903–8 (2013).
6. Keeling M, Rohani P. Modeling Infectious Diseases: In Humans and Animals. Princeton University Press; 2007.
7. Riolo, M. A. & Rohani, P. Combating pertussis resurgence: One booster vaccination schedule does not fit all. Proc Natl Acad Sci U S A 112, E472–7 (2015).
